# Supplementary material for: Klf9 is a key feedforward regulator of the transcriptomic response to glucocorticoid receptor activity
Source: Sci Rep. 2020 Jul 10;10:11415. doi: 10.1038/s41598-020-68040-z (PMC7351738; doi:10.1038/s41598-020-68040-z)
Supplement: Supplementary file 1 — Supplementary Figures [file 41598_2020_68040_MOESM1_ESM.pdf]

```
Human           1 MDSKESLTPGREENPSSVLAQERGDVMDFYKTLRGGATVKVSASSPSLAV     50
                        :..|..||...  ..||.:.:|:.|....|...:...|:.|:|.
Zfish           1 ------MDQGGLENGKK--RDERLNTLDYNKRATEGILPRRIQSTMSVAP     42

Human          51 AS---QSDSKQRRLLVDFPKGSVSNAQQPDLSKAVSLSMGLYMGETETKV     97
                  .|   |:....:.:..|.|.| :||:  |.|.:..|....::..::|.|:
Zfish          43 TSMVPQAGPMMQPVSGDIPNG-LSNS--PTLEEHTSSVSSIFGDDSELKL     89

Human          98 MGND-LGFPQQGQISLSSGETDLKLLEESIANLNRSTSVPENPKSSASTA    146
                  :|.: ....||..:..:.|:: |..||.|||:||       ||..|..:.
Zfish          90 LGKEQRALQQQTLVPFTLGDS-LSGLEASIADLN-------NPSPSMDSL    131

Human         147 VSAA-------PTEKEFPKTHSDVSSEQQHLKGQTG--TNGGNVKLYTTD    187
                  :...       .||...|....|:..:|... |..|  .:.||.||::
Zfish         132 IGGVDPNLFPLKTEDFSPMIKGDMDLDQDSF-GHIGKDVDVGNHKLFS--    178

Human         188 QSTFDILQDLEFSSGSPGKETNESPWRSDLLIDENCLLSPLAGEDDSFLL    237
                  .:|.|:|||.|. .|||          ||..:.::..||.:.  :|:.|.
Zfish         179 DNTLDLLQDFEL-DGSP----------SDFYVADDAFLSTIG--EDALLS    215

Human         238 EGNSNEDCKPLILPDTKPKIKDNGDL------VLSSPSNVTLPQVKTEKE    281
                  |..:|.|      .|:|..:..:..|      .||:.:...||.:|.||:
Zfish         216 ELPTNLD------RDSKAAVSGSNTLNGTASSSLSTANTSILPNIKVEKD    259

Human         282 DFIELCTPGVIKQEKLGTVYCQASFPGANIIGNKMSAISVHGVSTSGGQM    331
                  ..|:||||||||||..|..|||.        |...:.|::.||:||.||.
Zfish         260 SIIQLCTPGVIKQENTGASYCQG--------GLHSTPINICGVTTSSGQS    301

Human         332 YHYDMN--TASLSQQQDQKPIFNVIPPIPVGSENWNRCQGSGD-DNLTSL    378
                  :.:..:  ||.:..|:||||.||:..|:....:.|:|.||.|: ..:...
Zfish         302 FLFGNSSPTAVVGLQKDQKPDFNMYTPLTSSGDGWSRSQGFGNVSGMQQR    351

Human         379 GTLNFPGRTVFSNGYSSPSMRPDVSSPPSSSSTATTGPPPKLCLVCSDEA    428
                  .:|.|.     .|..|||..||: .|..:||:...|| ..|:||||||||
Zfish         352 ASLCFS-----KNFSSSPYSRPE-DSTATSSAGGKTG-THKICLVCSDEA    394

Human         429 SGCHYGVLTCGSCKVFFKRAVEGQHNYLCAGRNDCIIDKIRRKNCPACRY    478
                  |||||||||||||||||||||||||||||||||||||||||||||||||:
Zfish         395 SGCHYGVLTCGSCKVFFKRAVEGQHNYLCAGRNDCIIDKIRRKNCPACRF    444

Human         479 RKCLQAGMNLEARKTKKKIKGIQQATTGVSQETSEN------PGNKTIVP    522
                  ||||.|||||||||:|.|.:   ||...:.|::...      |..:.:||
Zfish         445 RKCLMAGMNLEARKSKSKAR---QAGKVIQQQSIPERNLPPLPEARALVP    491

Human         523 ATLPQLTPTLVSLLEVIEPEVLYAGYDSSVPDSTWRIMTTLNMLGGRQVI    572
                  ..:|||.||::|||:.|||:.|||||||::||::.|:|||||.|||||||
Zfish         492 KPMPQLVPTMLSLLKAIEPDTLYAGYDSTIPDTSVRLMTTLNRLGGRQVI    541

Human         573 AAVKWAKAIPGFRNLHLDDQMTLLQYSWMFLMAFALGWRSYRQSSANLLC    622
                  :|||||||:||||||||||||||||.||:|:|:|.||||||:..:.|:||
Zfish         542 SAVKWAKALPGFRNLHLDDQMTLLQCSWLFIMSFGLGWRSYQHCNGNMLC    591

Human         623 FAPDLIINEQRMTLPCMYDQCKHMLYVSSELHRLQVSYEEYLCMKTLLLL    672
                  |||||:|||:||.||.|.|||:.||.:|:|..|||||.|||||||.||||
Zfish         592 FAPDLVINEERMKLPYMSDQCEQMLKISNEFVRLQVSTEEYLCMKVLLLL    641

Human         673 SSVPKDGLKSQELFDEIRMTYIKELGKAIVKREGNSSQNWQRFYQLTKLL    722
                  ::|||||||||.:|||:||:|||||||||||||.||||||||||||||||
Zfish         642 NTVPKDGLKSQSVFDELRMSYIKELGKAIVKREENSSQNWQRFYQLTKLL    691

Human         723 DSMHEVVENLLNYCFQTFLDKTMSIEFPEMLAEIITNQIPKYSNGNIKKL    772
                  ||||::|..|||:||.||::|::|:||||||||||:||:||:.:|::|.|
Zfish         692 DSMHDLVGGLLNFCFYTFVNKSLSVEFPEMLAEIISNQLPKFKDGSVKPL    741

Human         773 LFHQK    777
                  |||||
Zfish         742 LFHQK    746
```

Figure S1. Needle amino acid sequence alignment of human and zebrafish glucocorticoid receptors. Methionines available as potential N-termini via alternative translation initiation are highlighted. Those in the zebrafish sequence were identified by the NetStart 1.0 algorithm. Those in the human GR are known to produce transcriptionally functional products (Lu and Cidlowski, 2005). The DNA binding domain is underlined.

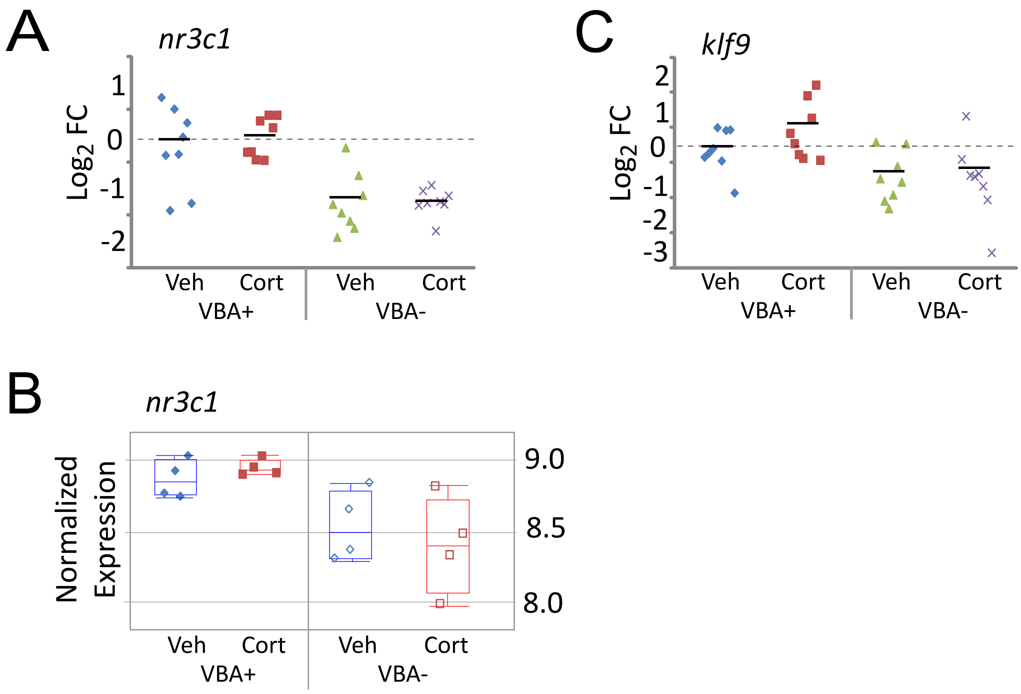

Figure S2. Expression of *nr3c1* and *klf9* is reduced in VBA- larvae. (A) Relative expression of *nr3c1* in individual VBA+ and VBA- larvae, measured by qRT-PCR; (B) normalized expression of *nr3c1* in each of the four biological replicates of the RNA-seq experiment (Fig. 1); (C) relative expression of klf9 in individual VBA+ and VBA- larvae, measured by qRT-PCR (same samples as in panel A).

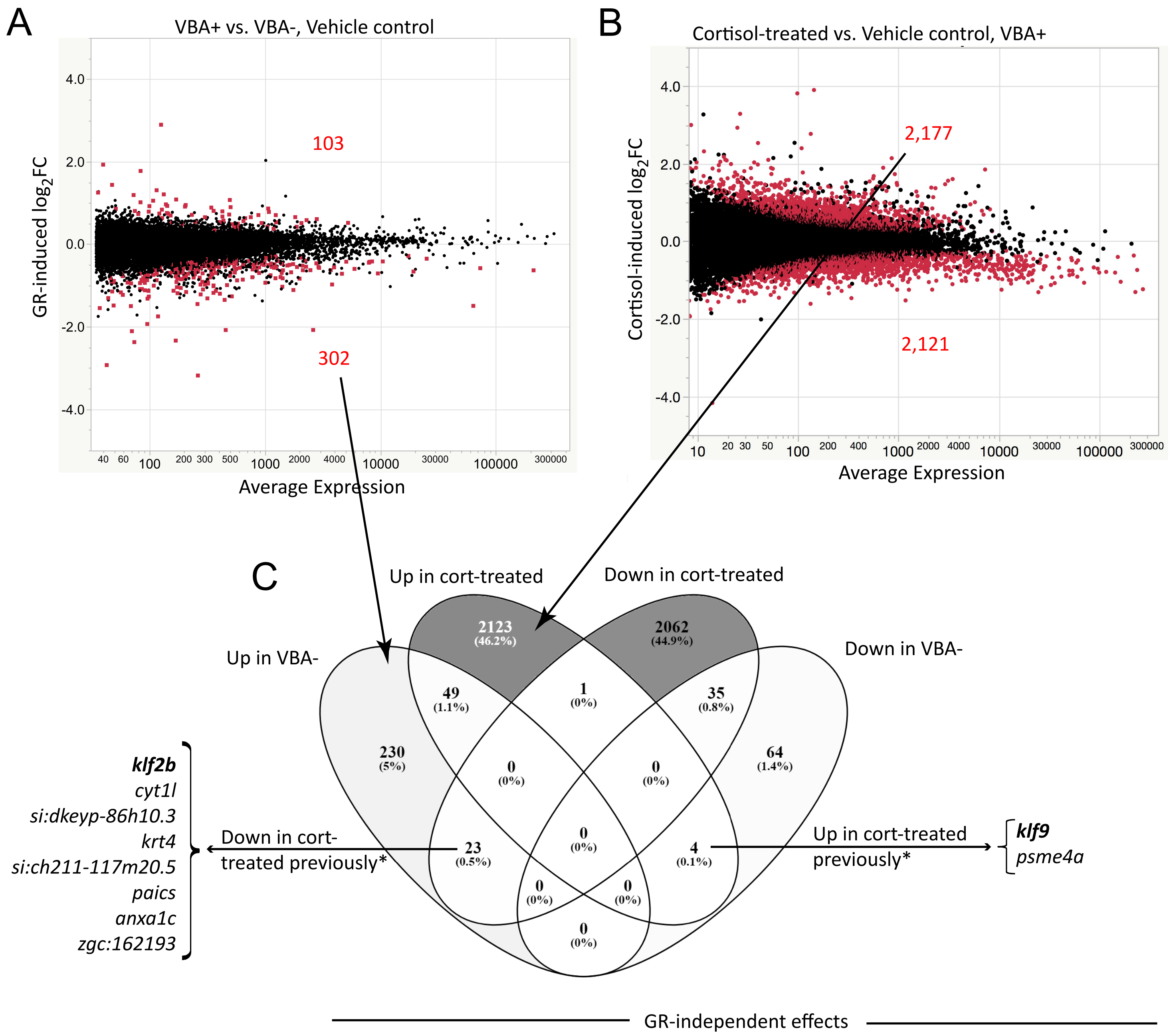

GR-independent effects

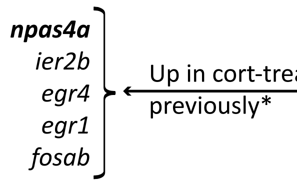

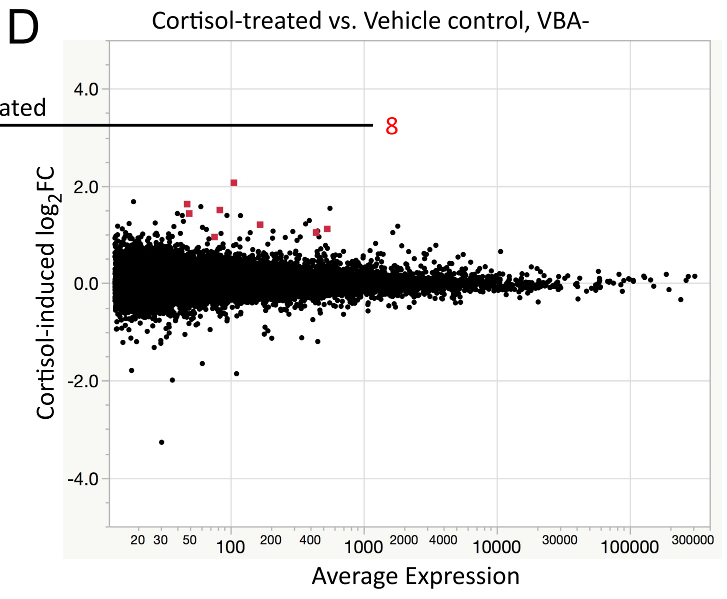

Figure S3. Transcriptomic effects of GR loss of function and chronic cortisol exposure in larvae containing or lacking a GR. (A) MA plot comparing gene expression in VBA+ and VBA- larvae developed under control conditions; (B) MA plot comparing gene expression in cortisol-treated VBA+ and vehicle-treated VBA+ larvae; (C) Venn diagram comparing the results of (A) and (B), and showing lists of genes* previously found to be down- or up-regulated by chronic cortisol (*Hartig et al., 2016); (D) MA plot comparing gene expression in cortisol-treated VBA- and vehicle-treated VBA- larvae. Diagram generated by Venny (https://bioinfogp.cnb.csic.es/tools/venny/).

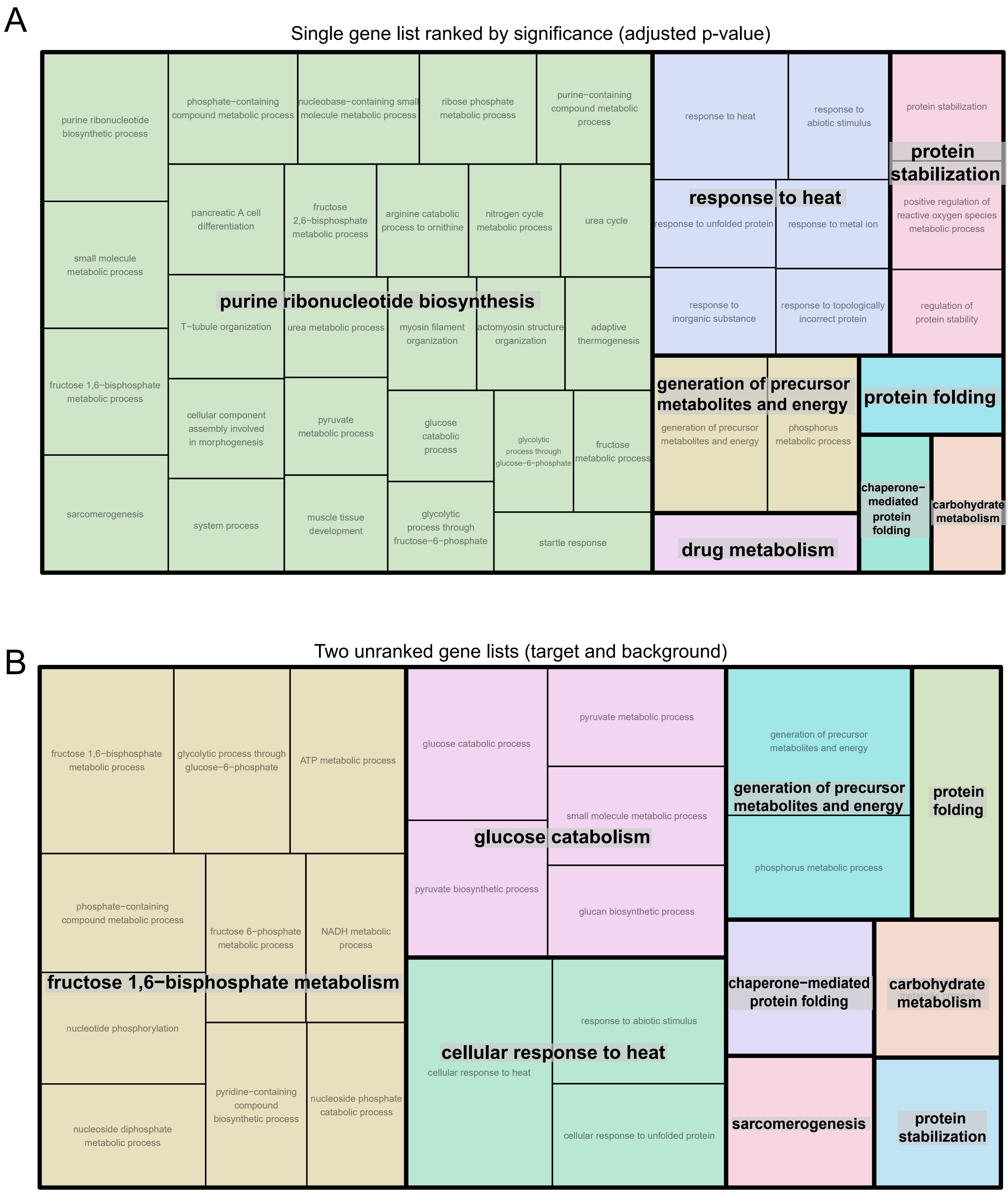

Figure S4. REVIGO Gene Ontology treemaps of processes upregulated by the GR under normal conditions, identified by GOrilla analysis of (A) single ranked gene list and (B) two unranked gene lists (target and background, with target genes having an adjusted p-value <0.05) obtained from differential gene expression analysis. Generated by REVIGO (http://revigo.irb.hr/).

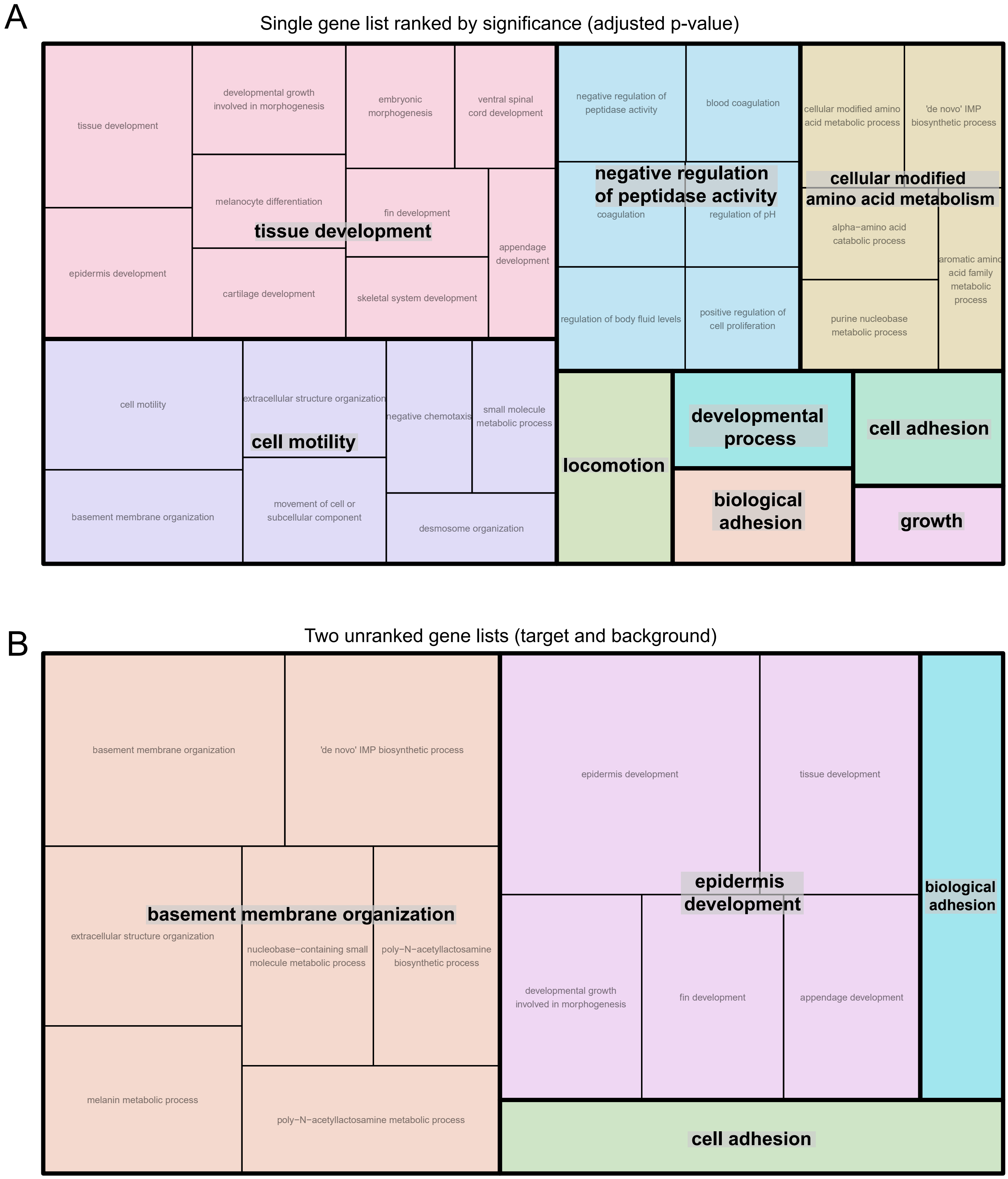

Figure S5. REVIGO Gene Ontology treemaps of processes downregulated by the GR under normal conditions, identified by GOrilla analysis of (A) single ranked gene list and (B) two unranked gene lists (target and background, with target genes having an adjusted p-value <0.05) obtained from differential gene expression analysis. Generated by REVIGO (http://revigo.irb.hr/).

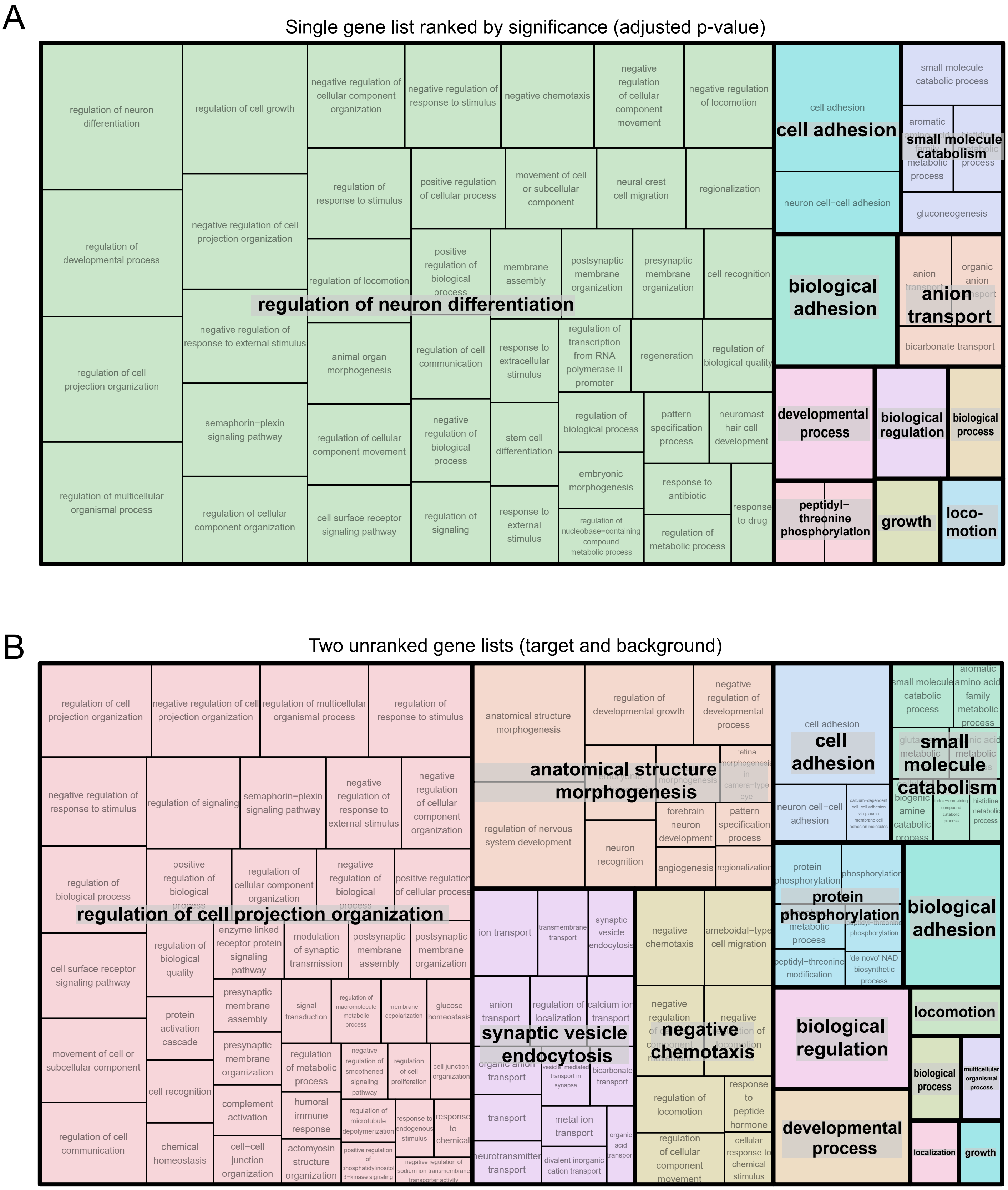

Figure S6. REVIGO Gene Ontology treemaps of processes upregulated by chronic cortisol treatment in larvae with a GR, identified by GOrilla analysis of (A) single ranked gene list and (B) two unranked gene lists (target and background, with target genes having an adjusted p-value <0.05) obtained from differential gene expression analysis. Generated by REVIGO (http://revigo.irb.hr/).

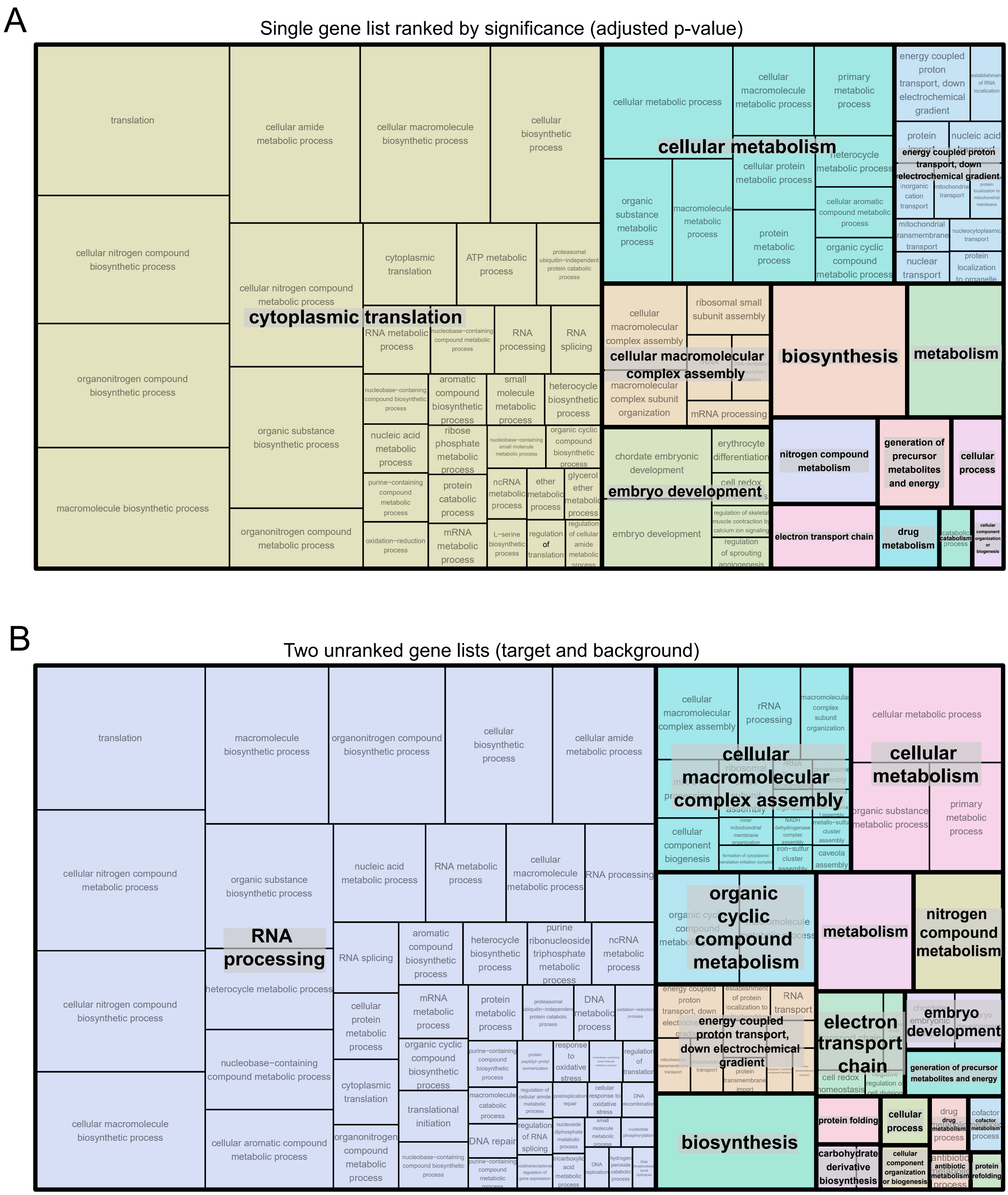

Figure S7. REVIGO Gene Ontology treemaps of processes downregulated by chronic cortisol treatment in larvae with a GR, identified by GOrilla analysis of (A) single ranked gene list and (B) two unranked gene lists (target and background, with target genes having an adjusted p-value <0.05) obtained from differential gene expression analysis. Generated by REVIGO (http://revigo.irb.hr/).

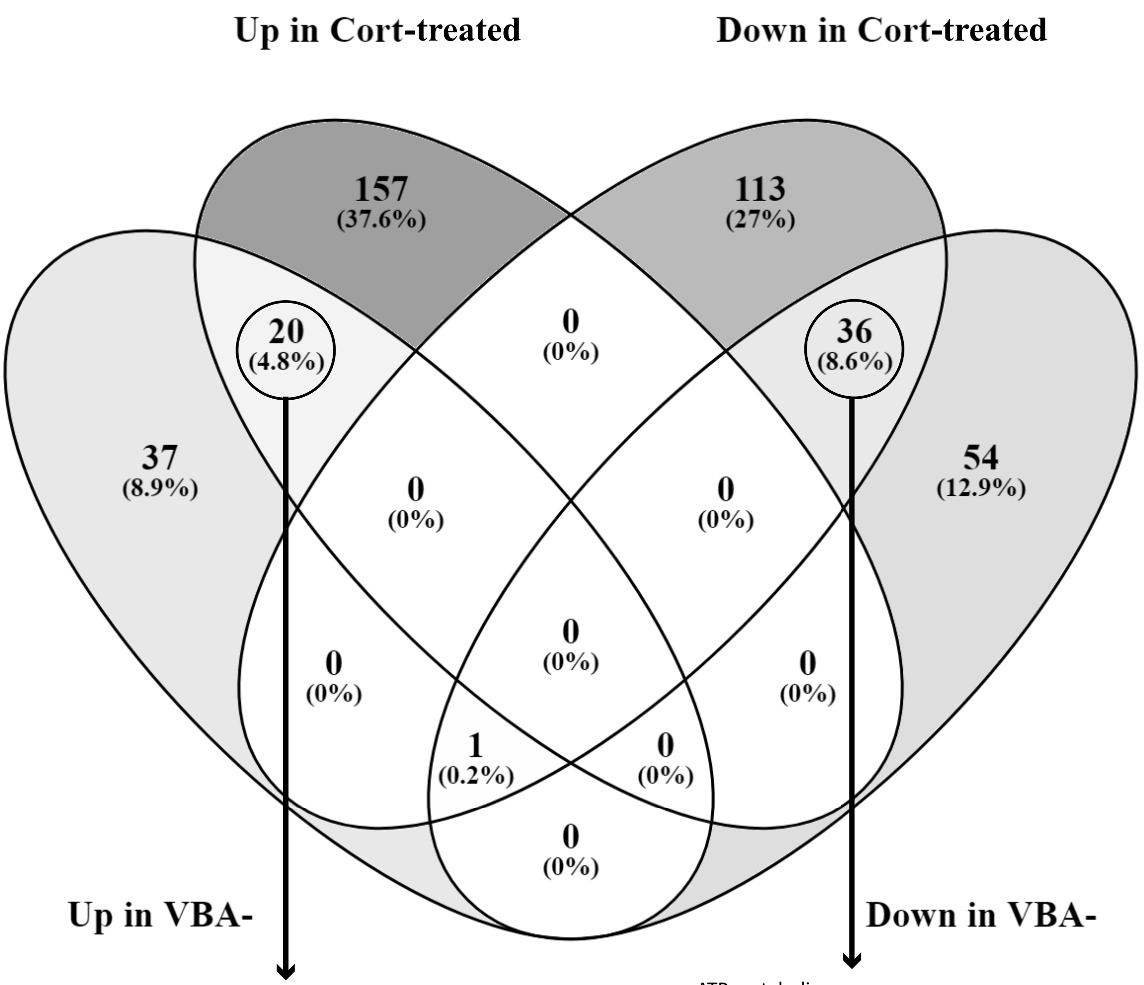

biological adhesion
cell adhesion
anatomical structure morphogenesis
developmental process
negative regulation of growth
negative chemotaxis
anatomical structure development
movement of cell or subcellular component
ameboidal-type cell migration
growth
developmental growth
cell migration
locomotion
cell motility
tissue development
cellular amino acid catabolic process
embryonic morphogenesis
alpha-amino acid catabolic process
aromatic amino acid family metabolic process
cell-substrate adhesion

ATP metabolic process
purine ribonucleoside triphosphate metabolic process
purine nucleoside triphosphate metabolic process
ribonucleoside triphosphate metabolic process
ATP biosynthetic process
purine ribonucleoside triphosphate biosynthetic process
purine nucleoside triphosphate biosynthetic process
nucleoside triphosphate metabolic process
purine nucleoside monophosphate biosynthetic process
purine ribonucleoside monophosphate biosynthetic process
purine nucleoside monophosphate metabolic process
purine ribonucleoside monophosphate metabolic process
nucleoside triphosphate biosynthetic process
ribonucleoside triphosphate biosynthetic process
ribonucleoside monophosphate biosynthetic process
nucleoside monophosphate biosynthetic process
ribonucleoside monophosphate metabolic process
purine ribonucleotide biosynthetic process
nucleoside monophosphate metabolic process
purine-containing compound biosynthetic process
purine nucleotide biosynthetic process
ribonucleotide biosynthetic process
drug metabolic process
ribose phosphate biosynthetic process
purine ribonucleotide metabolic process
nucleotide biosynthetic process
ribonucleotide metabolic process
purine-containing compound metabolic process
nucleoside phosphate biosynthetic process
purine nucleotide metabolic process
ribose phosphate metabolic process
nucleotide metabolic process
nucleoside phosphate metabolic process
nucleobase-containing small molecule metabolic process
organophosphate biosynthetic process

Figure S8. Venn diagram of numbers of biological processes shown by GOrilla analysis to be affected by presence or absence of the GR and by chronic cortisol treatment and overlap between them. Diagram generated by Venny (https://bioinfogp.cnb.csic.es/tools/venny/).

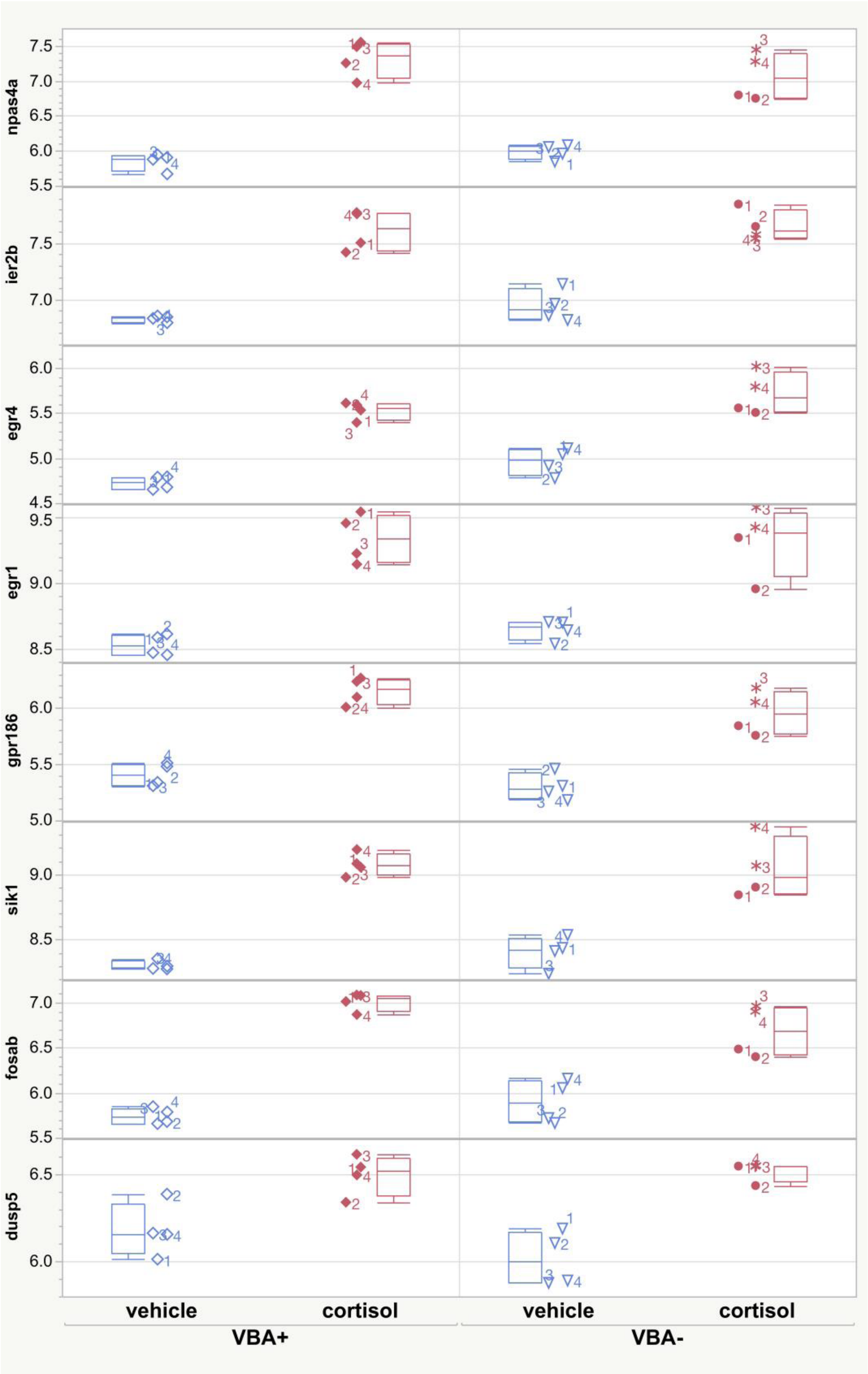

Figure S9. Expression levels of eight genes that are upregulated by chronic cortisol treatment in both VBA+ and VBA- larvae, measured by RNA-seq. Several of these genes (*npas4a, fosab, egr1, egr4, ier2b*) were found in our previously reported RNA-seq analysis to be upregulated in wild-type larvae treated chronically with cortisol (Hartig et al., 2016).

A

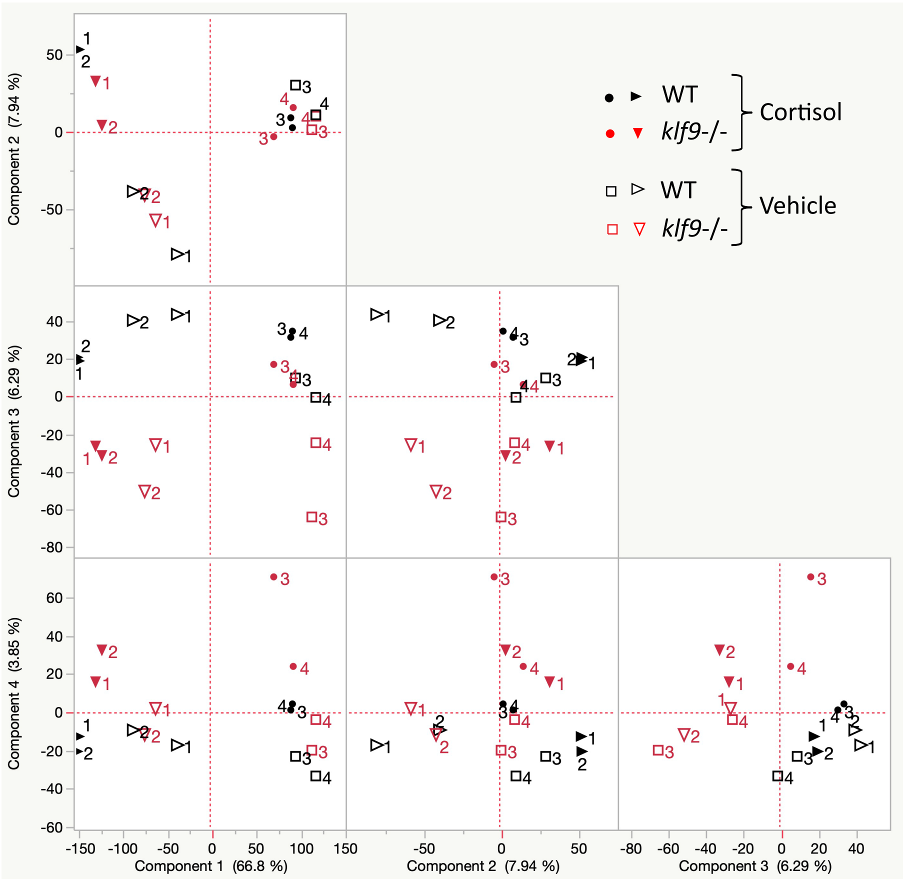

B

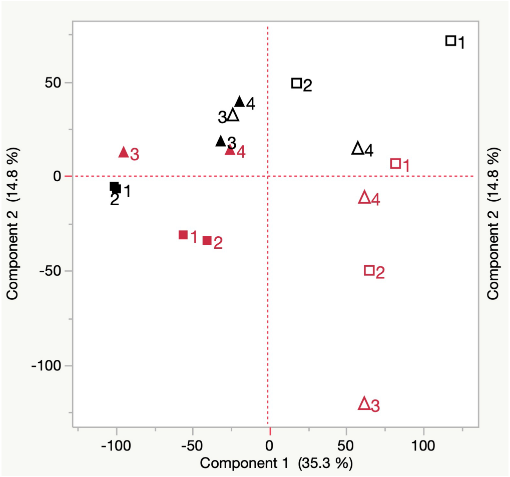

Figure S10. Principal component (PC) plots of the RNAseq data comparing transcriptomes of 5 dpf wild-type (WT) and *klf9*$^{-/-}$ larvae developed normally (vehicle) or with chronic cortisol treatment. (A) Plots of the first four PCs when all samples were rlog normalized as a group. (B) Plots of the first two PCs obtained when the data were rlog normalized independently for each day the samples were prepared (day 1, replicates 1 and 2; day 2, replicates 3 and 4).

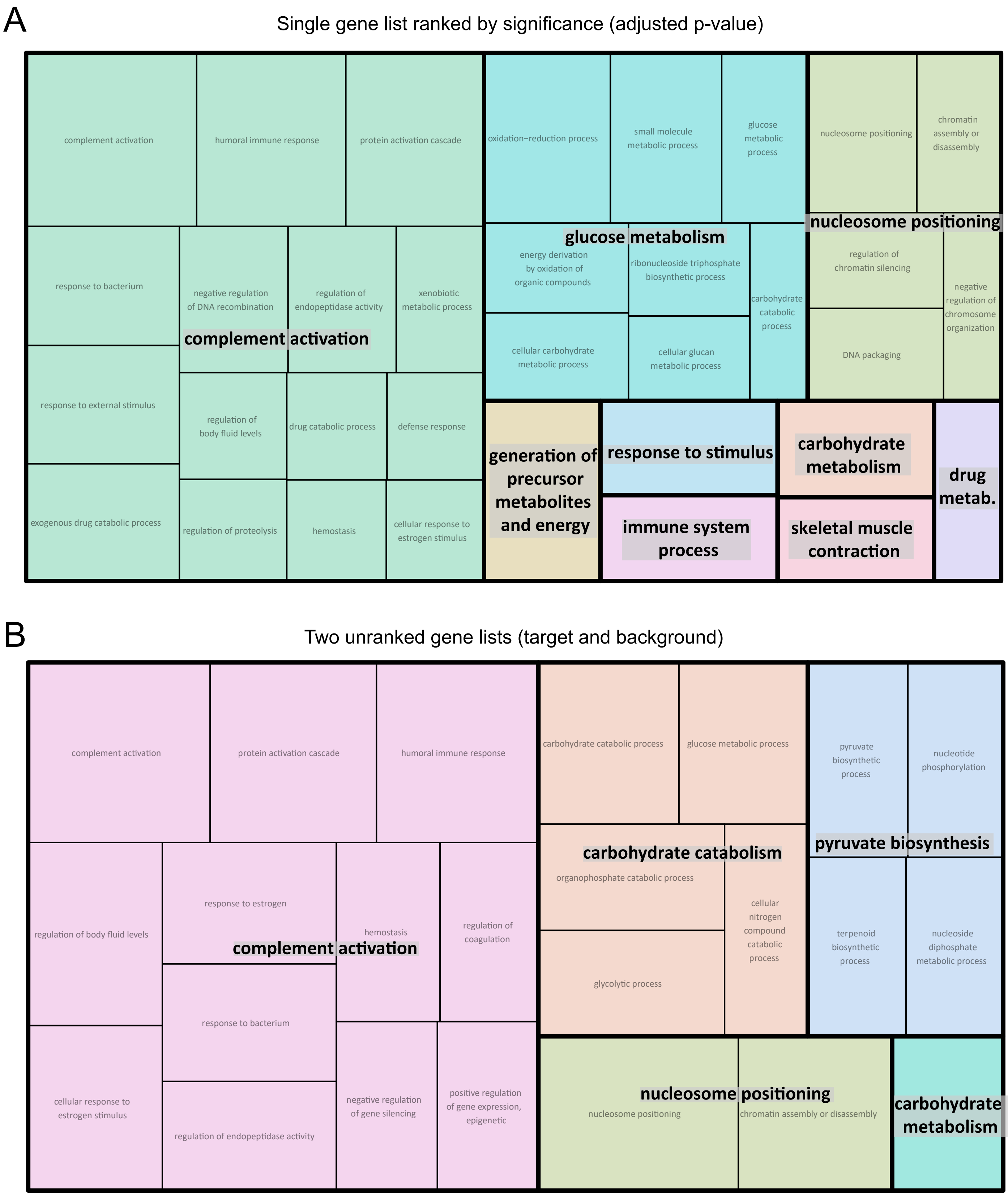

Figure S11. REVIGO Gene Ontology treemaps of processes upregulated by loss of Klf9 function, identified by GOrilla analysis of (A) single ranked gene list and (B) two unranked gene lists (target and background, with target genes having an adjusted p-value <0.05) obtained from differential gene expression analysis. Generated by REVIGO (http://revigo.irb.hr/).

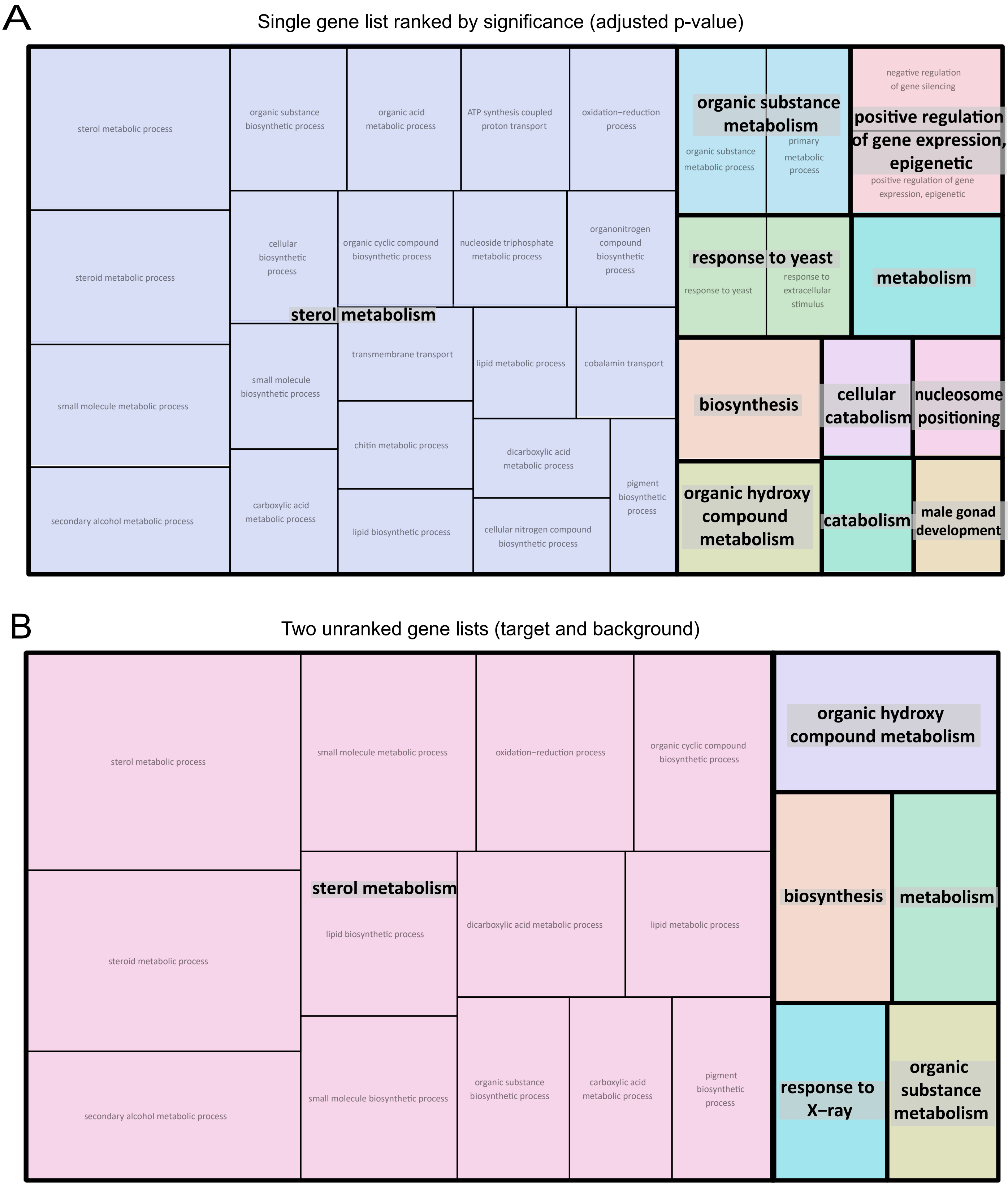

Figure S12. REVIGO Gene Ontology treemaps of processes downegulated by loss of Klf9 function, identified by GOrilla analysis of (A) single ranked gene list and (B) two unranked gene lists (target and background, with target genes having an adjusted p-value <0.05) obtained from differential gene expression analysis. Generated by REVIGO (http://revigo.irb.hr/).

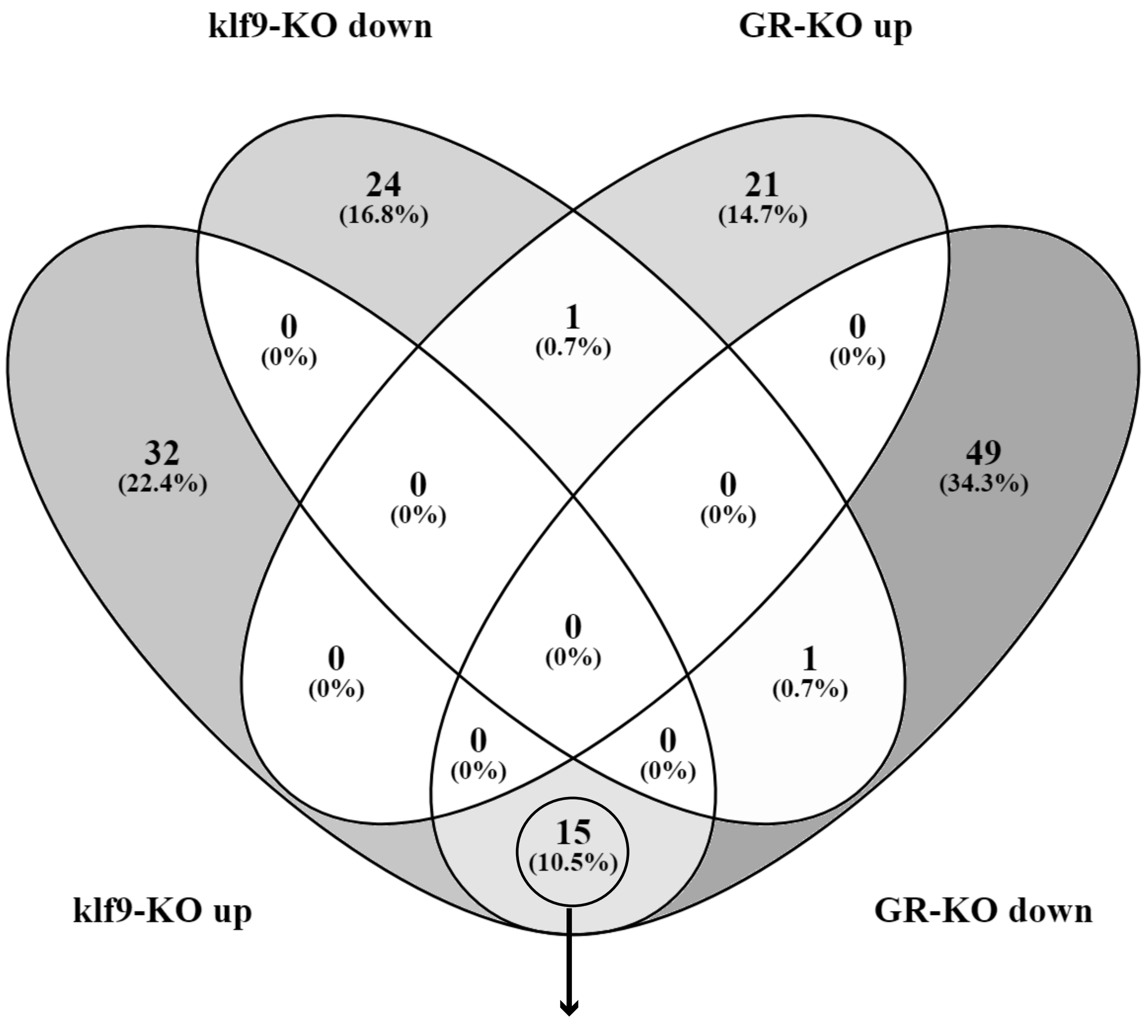

ATP generation from ADP
glycolytic process
pyruvate biosynthetic process
ADP metabolic process
nucleoside diphosphate phosphorylation
nucleotide phosphorylation
ribonucleoside diphosphate metabolic process
purine ribonucleoside diphosphate metabolic process
purine nucleoside diphosphate metabolic process
pyruvate metabolic process
nucleoside diphosphate metabolic process
carbohydrate metabolic process
hexose catabolic process
pyridine nucleotide biosynthetic process
nicotinamide nucleotide biosynthetic process

Figure S13. Venn diagram of numbers of biological processes shown by GOrilla analysis to be affected by presence or absence of the GR and presence or absence of Klf9 and overlap between them. Diagram generated by Venny (https://bioinfogp.cnb.csic.es/tools/venny/).

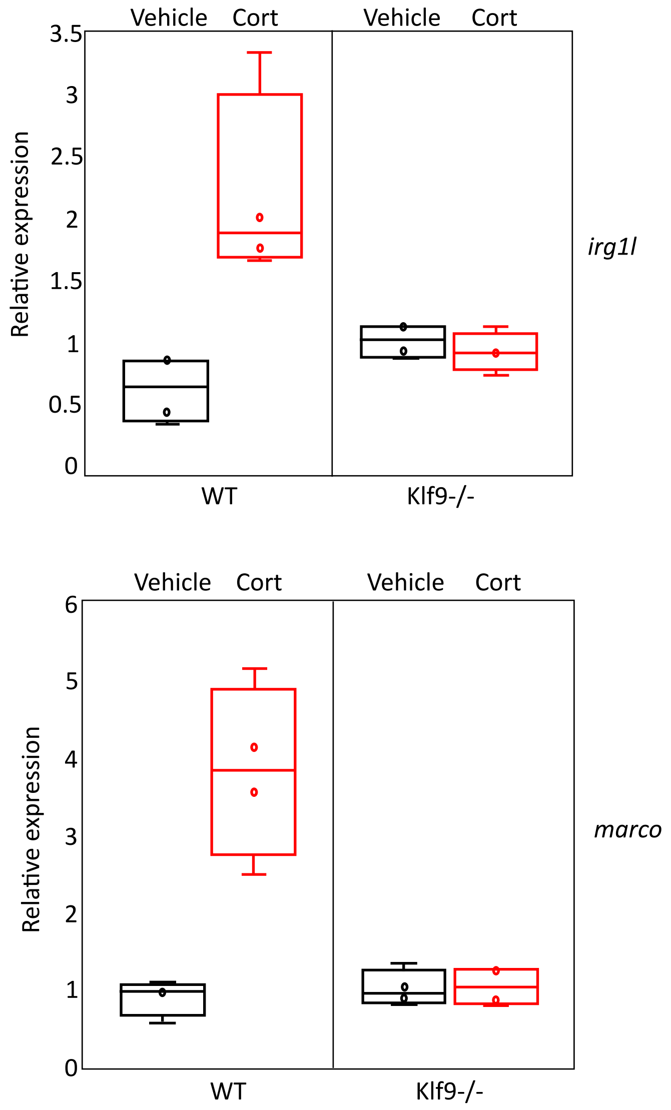

Figure S14. Relative expression levels of *irg1l* and *marco* measured by qRT-PCR, in the same RNA samples that were subjected to RNA-seq (see Fig. 4).

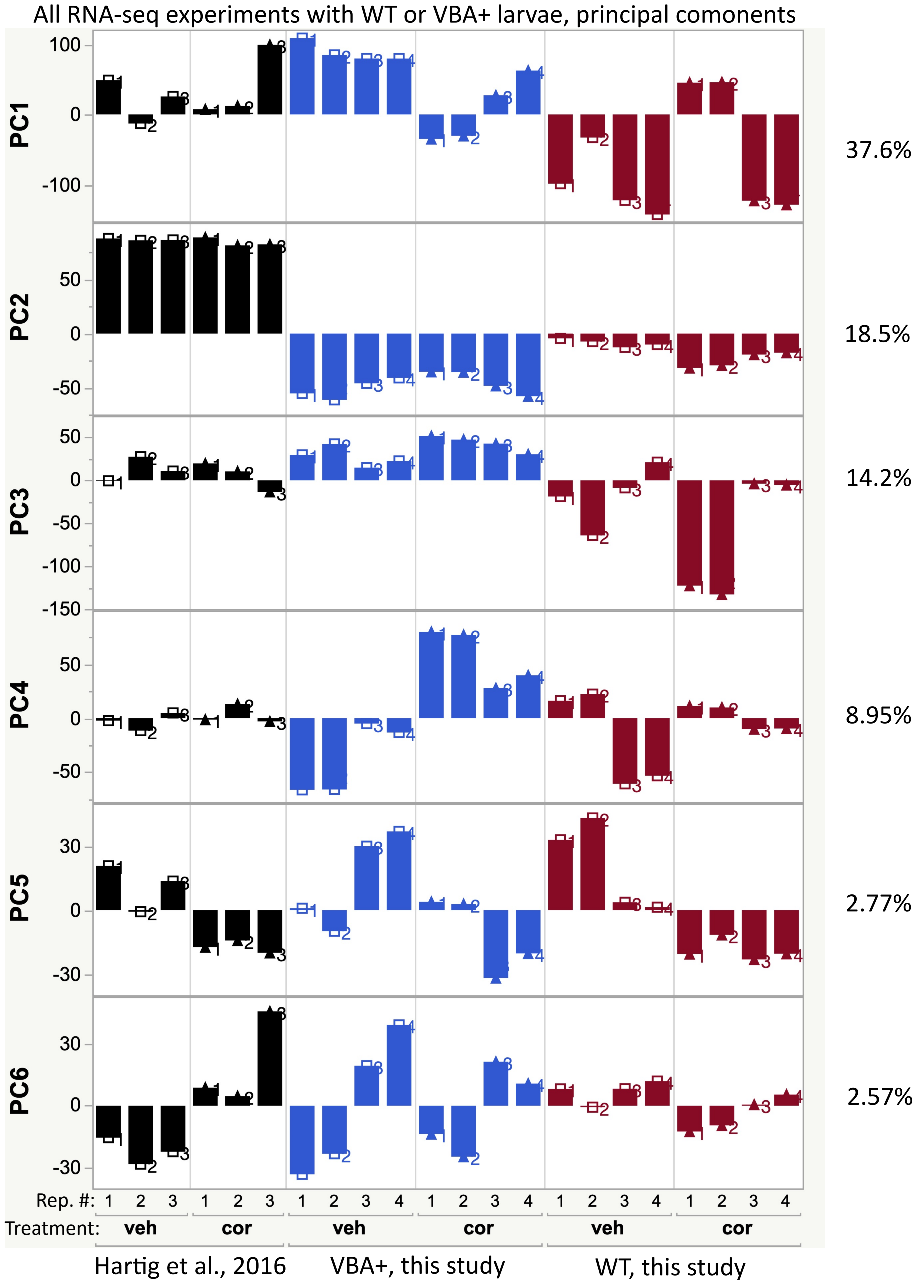

Figure S15. Principal components of the variance in gene expression across three RNA-seq experiments examining the transcriptomic effects of chronic cortisol treatment.
