# Supplementary material for: Klf9 is a key feedforward regulator of the transcriptomic response to glucocorticoid receptor activity
Source: Sci Rep. 2020 Jul 10;10:11415. doi: 10.1038/s41598-020-68040-z (PMC7351738; doi:10.1038/s41598-020-68040-z)
Supplement: Supplementary file 19 — Supplementary legends [file 41598_2020_68040_MOESM19_ESM.docx]

**Supplementary Table Legends**

Table S1a: Biological Processes associated with genes differentially expressed in VBA+ vs. VBA- larvae (vehicle controls), identified by GOrilla analysis of single ranked lists

Table S1b: Biological Processes associated with genes differentially expressed in VBA+ vs. VBA- larvae (vehicle controls), identified by GOrilla analysis of two unranked lists

Table S2a: Biological Processes associated with genes differentially expressed in cortisol-treated vs. vehicle treated VBA+ larvae, identified by GOrilla analysis of single ranked lists

Table S2b: Biological Processes associated with genes differentially expressed in cortisol-treated vs. vehicle treated VBA+ larvae, identified by GOrilla analysis of two unranked lists

Table S3: Genes from intersections shown in Supplementary Figure S3C.

Table S4: Biological Processes associated with PC1 shown in Supplementary Figure S10, identified by GOrilla analysis of single ranked list of genes

Table S5a: Biological Processes associated with genes differentially expressed in *klf9*^-/-^ vs. wild type larvae (vehicle controls), identified by GOrilla analysis of single ranked lists

Table S5b: Biological Processes associated with genes differentially expressed in *klf9*^-/-^ vs. wild type larvae (vehicle controls), identified by GOrilla analysis of two unranked lists

Table S6: List of the 408 genes upregulated by cortisol treatment in wild-type but not *klf9*^-/-^ larvae

Table S7: Biological Processes associated with genes upregulated by cortisol in wild-type and *klf9*^-/-^ larvae, identified by GOrilla analysis of two unranked lists

Table S8: Biological Processes associated with PC5 shown in Figure 5 and Supplementary Figure S15, identified by GOrilla analysis of single ranked list of genes

Table S9: Biological Processes associated with PC2 shown in Supplementary Figure S15, identified by GOrilla analysis of single ranked list of genes

Table S10: Biological Processes associated with PC1 shown in Supplementary Figure S15, identified by GOrilla analysis of single ranked list of genes

Table S11: Annotated list of the 149 genes found to be upregulated by cortisol treatment in 2 or more of the RNA-seq experiments

Table S12: Biological Processes associated with the 149 genes upregulated by cortisol treatment in 2 or more of the RNA-seq experiments, identified by GOrilla analysis of two unranked lists

Table S13: HOMER motif enrichment analyses

Table S14: Statistical comparisons for the 149 genes found to be upregulated by cortisol treatment in 2 or more of the RNA-seq experiments
